# Comprehensive exploration of the role of multimodal programmed cell death- associated lncRNAs in the prognosis and immunity of glioma

**DOI:** 10.3389/fimmu.2026.1765882

**Published:** 2026-05-14

**Authors:** Shuaishuai Wu, Xiangji Meng, Yanran Hu, Qi Wang, Yanfen Yao, Haiyan Jia, Zhen Ma, Yang Liu, Zhongxu Sun, Yahu Bai, Haixia Jin, Lingzhi Li, Changli Wang

**Affiliations:** 1 Department of Intensive Care Medicine, Shandong Provincial Third Hospital, Shandong University, Jinan, China; 2 Department of Neurosurgery, Shandong Cancer Hospital and Institute, Shandong First Medical University and Shandong Academy of Medical Sciences, Jinan, China; 3 Department of Pathology, Shandong Cancer Hospital and Institute, Shandong First Medical University and Shandong Academy of Medical Sciences, Jinan, China; 4 Department of Paediatrics, Shandong Provincial Hospital Affiliated to Shandong First Medical University, Jinan, China

**Keywords:** glioma, immune function, lncRNA, prognosis, programmed cell death

## Abstract

**Introduction:**

Gliomas have an inferior prognosis and urgently require new biomarkers and treatment strategies. Although some studies have explored the association between specific categories of programmed cell death (PCD) and glioma, research on the combined mechanism of PCD and its contribution to glioma development remains insufficient. This study aims to systematically investigate the role of lncRNAs in PCD in relation to the prognosis of glioma.

**Methods:**

A prognostic risk model based on key lncRNAs was screened and constructed from multiple PCD pathways through bioinformatics analysis. And conduct a comprehensive analysis of its immune function and its effects. To validate the model, we selected the key molecule AC092718.4 for functional experiments.

**Results:**

Our research indicates that AC092718.4 significantly alters the proliferative and invasive capabilities of glioma cells. Animal experiments further confirmed that it can promote tumor growth.

**Conclusion:**

This study successfully constructed a robust PCD-related lncRNA prognostic model, providing a new tool for risk stratification in glioma patients and laying the groundwork for developing precision treatment strategies based on programmed cell death.

## Introduction

Central nervous system (CNS) tumors, classified into primary and metastatic types based on their origin, represent one of the leading causes of morbidity and mortality worldwide (1). As a primary CNS tumor, glioma exhibits low overall incidence but poses significant treatment challenges due to its malignant biological behavior (high heterogeneity and invasiveness) (2). For instance, despite standard therapies, the median survival for glioblastoma remains approximately 14 months (3). Additionally, another category of malignant tumors in the CNS comprises brain metastases (BMs), which occur at a significantly higher rate than primary tumors. Statistics indicate that nearly half of all cancers invade the brain to varying degrees (4), primarily including NSCLC (45%), BC (15%), and melanoma (10%) among all brain metastases. However, the overall prognosis for CNS metastases is often extremely poor (5). Despite advances in diagnostic and therapeutic technologies, the median survival for these patients remains unsatisfactory. This not only inflicts immense physical and psychological suffering on patients but also imposes sustained, heavy economic burdens and caregiving responsibilities on their families and society (6). Hence, the early recognition and diagnosis of molecular biomarkers with prognostic predictive value in clinical practice, along with the active exploration and application of innovative treatment strategies that can fundamentally improve long-term outcomes, are urgently needed.

Programmed cell death (PCD) is a genetic-regulated, actively initiated, and well-ordered process of cell death that encompasses multiple forms, including apoptosis, programmed necrosis, pyroptosis, ferroptosis, and cupric-mediated cell death. It maintains tissue homeostasis by eliminating abnormal cells within the body (7). Multiple studies indicate that PCD can participate in tumorigenesis and progression, with mutations in genes or receptors within its pathways potentially driving the transformation of healthy cells into malignancies (8). Concurrently, researchers have discovered that interfering with PCD-related pathways can inhibit tumor progression, offering novel therapeutic directions for targeted treatments (9). Current research on glioblastoma multiforme is focused explicitly on activating PCD-related pathways to explore new therapeutic strategies (10, 11). For instance, studies have found that the tyrosine kinase inhibitor (Apatinib) can inhibit tumor proliferation in glioblastoma cells by inducing ferroptosis (11). Additionally, another anti-tumor drug (Roxastat) can stimulate ferroptosis by enhancing the HIF signaling pathway, thereby preventing the growth of glioblastoma cells (12). Similarly, PCD plays a vital role in the emergence and progression of certain brain metastases. For example, the absence of TAK1 increases RIPK3 expression levels in endothelial cells, thereby enhancing their apoptotic potential and tumor cell metastasis capacity (13).In addition, phosphorylation of the necroptosis marker MLKL has been detected in the necrotic areas of BC samples and mouse models (14). In summary, these findings indicate that PCD occurs throughout all stages of tumorigenesis and the pathogenesis of metastasis. Therefore, a thorough investigation of the complex molecular pathways underlying programmed cell death is crucial for innovating targeted therapies for central nervous system tumors.

Although PCD pathways—such as apoptosis, pyroptosis, ferroptosis, necroptosis, and autophagy—differ in their mechanisms of action, they do not operate in isolation. Instead, they intersect through shared signaling hubs and regulatory networks, collectively maintaining tissue homeostasis and influencing tumor progression (15). For example, excessive reactive oxygen species can simultaneously trigger ferroptosis and necrotic apoptosis (16); mitochondrial dysfunction contributes to intrinsic apoptosis and regulates autophagy (17); and lysosomal membrane permeabilization can activate pyroptosis and autophagic cell death (18). Upstream key transcription factors such as p53, NF-κB, and HIF-1α can simultaneously regulate multiple PCD pathways, forming complex transcriptional networks (19). In the tumor microenvironment, tumor cells often evade death pressures induced by a single pathway by switching between different PCD modes, leading to treatment resistance. Therefore, focusing on a single pathway makes it difficult to fully elucidate its role in glioma. This study proposes an integrative analytical strategy that treats multiple PCD pathways as a synergistically regulated network system. By systematically screening relevant lncRNAs, this approach aims to accurately reflect the death regulatory status of tumor cells within complex microenvironments, thereby better capturing the clinical realities of tumor heterogeneity and pathway compensation.

With the advancement of molecular biology research, lncRNAs have been recognized as new players in genetic regulation, playing an increasingly prominent role in regulating PCD within CNS tumors. LncRNAs have been demonstrated to extensively participate in cellular processes across multiple levels, including gene imprinting, chromatin remodeling, transcription, and post-transcriptional regulation, thereby assuming critical roles in tumour initiation and growth (20). In gliomas, studies indicate that LINC00942 promotes the expression of the transcription factor SOX9 by interacting with two key metabolic enzymes (TPI1 and PKM2), consequently enabling tumor cell stem cell properties and resistance to TMZ (21); lncRNA INHEG, highly expressed in glioblastoma stem cells, promotes the self-renewal and tumorigenic potential of these stem cells by regulating 2’-O methylation of rRNA (22). Furthermore, the regulatory functions of lncRNAs extend to the tumor microenvironment and metastasis processes. Research indicates that lncRNA-CCRR upregulates CX43 expression, thereby promoting gap junction formation in breast cancer BMs and accelerating its progression (23). Another study revealed that LINC00482 enhances NSCLC brain metastasis by inducing M2 polarization of microglia and influencing tumor cell migration through modulation of the miR-142-3p/TGF-β1 Axial Pathway. Moreover, blocking signaling mediated by tumor-derived LINC00482 or its extracellular vesicles may represent a potential therapeutic target for inhibiting BMs in NSCLC (24). Furthermore, lncRNAs have been demonstrated to influence tumor invasion and distant implantation capacity by regulating epithelial-mesenchymal transition or angiogenesis, which is crucial for understanding the formation of brain metastasis (25). Therefore, this study examines the mechanisms of action of lncRNAs associated with PCD in glioma prognosis assessment and pathogenesis, explore efficient biomarkers for early identification and risk stratification, and lay a solid theoretical foundation for developing novel, precision-targeted therapeutic approaches based on programmed cell death.

## Materials and methods

### Data and screening criteria

We downloaded glioma data (GBM and LGG) and normal brain tissue RNA transcriptomic data from the The Cancer Genome Atlas (TCGA) and Genotype-Tissue Expression (GTEx) websites (698 glioma samples and 1, 152 normal human brain samples, respectively). We then converted the FPKM values to TPM values in a synthetic matrix using the R packages ‘data.table’, ‘tibble’, ‘dplyr’, and ‘tidyr’. As a result, we obtained two synthetic data matrices. The counts matrix was used exclusively to identify differentially expressed lncRNAs, while the TPM matrix was used for other analyses. To minimize statistical bias in this analysis, glioma patients with missing overall survival (OS) data or short survival (<30 days) were excluded. Combining relevant clinical information, the data were randomly split into training and validation risk groups (7:3 ratio) using the Strawberry Perl and caret R packages.

### Identification of PCD-related lncRNAs

PCD-related genes (apoptosis, programmed necrosis, necrotic apoptosis, autophagy, pyroptosis, ferroptosis) were obtained from the GeneCards database (**Supplementary Material 1**). Next, we used a Venn diagram to remove duplicate genes, resulting in a set of 21 genes. We then employed the Wilcoxon method (FDR< 0.05, Log2 FC > 1) to screen for genes with significant differences in expression levels between glioma and normal tissues. After removing genes without significant differences, the remaining genes constituted the PCD-related differentially expressed genes (19 genes). Finally, we performed co-expression analysis on the PCD-related differentially expressed genes (19 genes) and lncRNAs, identifying PCD-related lncRNAs (Pearson correlation coefficient > 0.4, p< 0.001).

### Establishment of a prognostic risk model

First, key lncRNAs identified as prognostic factors for glioma were determined using Lasso and Cox regression analyses (p< 0.05). Specifically, the Lasso regression analysis was subjected to 10-fold cross-validation and run for 1, 000 iterations. Each iteration included 1, 000 random resampling runs to prevent overfitting. Subsequently, the Lasso regression results were incorporated into a multivariate Cox regression analysis, which identified eight PCD-associated lncRNAs and generated a Risk Rcore (RS) formula:


$$\text{risk}\;\text{score=}\sum_{\text{i=l}}^{n}\text{coef}\;\text{PCD}\;\text{LncS}i\text{g}i\;*\;\mathrm{EXP}\;\mathrm{PCD}\;\mathrm{LncS}\text{i}gi$$

In this “risk score” formula, “coef PCD Lncsigi” represents the coefficient values, specifically the regression coefficients for the eight prognostic lncRNAs derived from multivariate regression analysis. In the formula, “EXP PCDLncSigi” represents the expression levels of the 8 PCD-related lncRNAs. Using the risk score formula, we can calculate a risk value for each patient. After obtaining the risk values for all patients, the median value is used to determine the patients’ risk levels (classified into high- and low-risk groups).

### Immune function and drug analysis

Single-sample GSEA was employed to evaluate the immune cell activity and pathways of the predicted factors, followed by analysis of the fraction of infiltrating immune cells in tumor samples. Measure the levels of multiple drugs in glioma samples to evaluate relevant immune-related therapeutic agents.

### Cell transfection

We used two glioma cell lines (U87 and U251). Cells in good growth condition were infected with a lentiviral vector carrying the AC092718.4 sequence and a lentiviral vector carrying shRNA targeting AC092718.4 (Shanghai Jike Gene Medical Technology Co., Ltd.). Subsequently, the cells were transferred to an incubator for lentiviral transduction experiments. Cells in good growth condition were harvested to prepare a cell suspension (5 × 10^4^ cells/mL), which was then seeded into wells according to experimental groups at 2 mL per well. Using a multiplicity of infection (MOI) of 20, the corresponding viral dose was added to each well. Approximately 2–3 days after infection, stable cell lines were selected using puromycin.

### CCK-8 cell proliferation assay

Prepare a cell suspension with a concentration of 2 × 10^4^ cells/mL. Add CCK-8 reagent (10 μL/well) to wells at different periods. Wells containing cell-free medium served as blank controls. Continue culturing in the cell incubator.

### Colony formation and transwell invasion assay

Seed U87 and U251 experimental and control group (nc) cells at a density of 1000 cells per well into corresponding 6-well plates and incubate in the cell culture incubator. Perform statistical analysis of colony counts—Pre-permeabilize Transwell chambers (Corning Costar, 8μm) with Matrigel. Resuspend transfected control and experimental group cells, and cells undergo fixation, staining, and counting procedures.

### Apoptosis assay

Digest the virus-infected U87 cells with EDTA-free trypsin, then transfer them to a centrifuge tube. Centrifuge to pellet the cells. Resuspend the cells in pre-chilled PBS (1 mL), centrifuge again, and discard the supernatant. Dilute the binding buffer with deionized water. Resuspend the cells in 1× binding buffer at a concentration of 4–6 × 10^6^ cells/mL. Using a pipette, draw up 100 µL of the cell suspension. Add Annexin V-PE and PI as directed, mix well, and incubate at room temperature in the dark for 5 minutes. Add 10 µL of PI and 400 µL of PBS, then perform flow cytometry analysis.

### Nude mouse *in vivo* tumor formation assay

Four-week-old female nude mice were randomly divided into two groups of six mice each. U87 cells were administered to either the control group or the experimental group. Each nude mouse received an intracerebral injection of 1 × 10^5^ cells. *In vivo* imaging was performed weekly to monitor tumor growth, and the experiment concluded after 28 days. The Ethics Committee of Shandong First Medical University Affiliated Cancer Hospital approved this study.

### Flow cytometric analysis of tumor-infiltrating immune cells

An intracerebral tumor model (U87 cells) was established in C57BL/6 mice using a nude mouse orthotopic tumor model protocol. Tumor tissues (control group and AC092718.4 overexpression group) were processed using mechanical dissociation and enzymatic digestion to prepare single-cell suspensions. Tumor-infiltrating immune cells were isolated using density gradient centrifugation or appropriate immune cell enrichment methods. Subsequently, flow cytometric analysis was performed using specific antibodies to detect immune cell subsets, including CD8^+^ T cells and the exhaustion marker TIM-3. Data were collected using a flow cytometer, and the proportions and phenotypes of immune cells across different groups were compared and analyzed.

### Statistical analysis

R software (version 4.1.2) was used for this study. Results from clonogenic and trans-epithelial invasion assays were analyzed using ImageJ software. Statistical significance between two groups was assessed using GraphPad Prism 8.0.

## Results

### Identification of PCD-related lncRNAs

**Figure 1:**
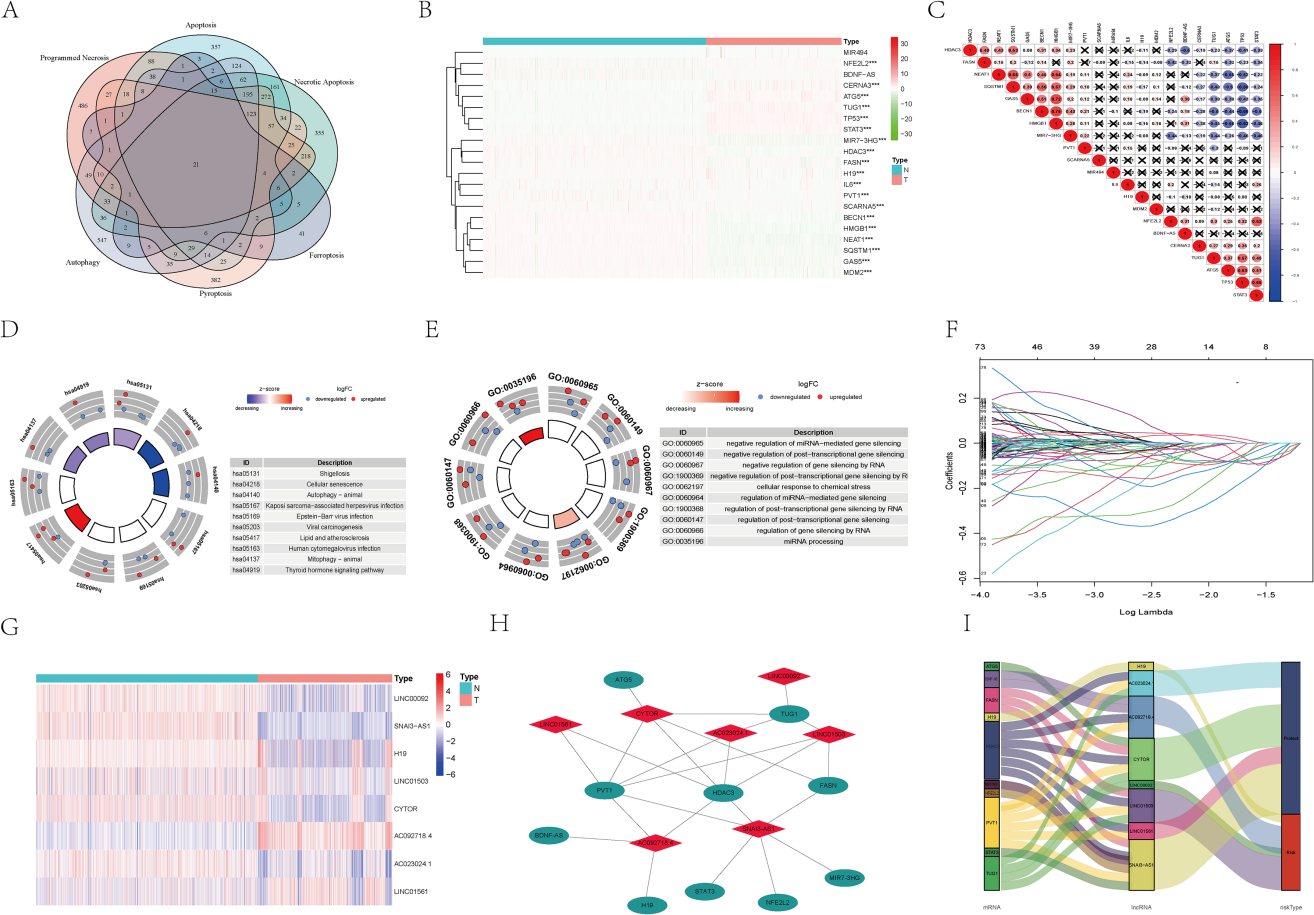
Screening of lncRNAs related to programmed cell death. **(A)** Screen for identical genes present in apoptosis, programmed necrosis, necrotic apoptosis, autophagy, pyroptosis, and ferroptosis. **(B)** Heatmap of DEGs related to programmed cell death. **(C)** Correlation analysis of DEGs related to programmed cell death. **(D)** KEGG analysis of DEGs related to programmed cell death. **(E)** GO analysis of DEGs related to programmed cell death. **(F)** Lasso regression screening for prognostic lncRNAs related to programmed cell death. **(G)** Heatmap of lncRNAs associated with programmed cell death prognosis. **(H)** The correlation between lncRNA and genes in the prognosis of programmed cell death. **(I)** Sankey diagram of lncRNAs associated with programmed cell death prognosis. N, Normal; T, Tumor. (***P< 0.001).

Based on the GeneCards database, genes associated with PCD (apoptosis, programmed necrosis, necrotic apoptosis, autophagy, pyroptosis, ferroptosis) were identified. A Venn diagram analysis screened out 21 common genes (**Figure 1A**). Subsequently, differential expression analysis between glioblastoma and normal brain tissue identified 19 PCD-associated DEGs (**Figure 1B**; MIR494 and BDNF-AS showed no differential expression). Furthermore, exploring intrinsic correlations among PCD-related genes revealed that BECN1 exhibits significant positive and negative correlations with HMGB1 and TP53, respectively (**Figure 1C**). Functional enrichment analysis revealed that DEGs were predominantly enriched in cell death and transcription-related pathways, including cellular senescence, autophagy, mitophagy, and transcriptional gene silencing by RNA (**Figures 1D, E**). Subsequently, Lasso regression analysis identified 568 PCD-associated lncRNAs (**Supplementary Material 1**). Multivariate Cox regression selected eight lncRNAs with optimal predictive correlation: SNAI3-AS1, AC023024.1, LINC01561, CYTOR, H19, AC092718.4, LINC01503, and LINC00092 (**Figures 1F, G**; **Table 1**). Cytoscape visualization of lncRNA-mRNA correlations (**Figure 1H**) revealed that SNAI3-AS1 showed stronger correlations with six PCD-related genes (HDAC3, FASN, STAT3, NFE2L2, PVT1, and MIR7-3HG); CYTOP co-expressed with 5 related genes (ATG5, TUG1, PVT1, HDAC3, FASN); AC092718.4 co-expressed with 4 related genes (PVT1, HDAC3, BDNF-AS, H19). In addition, SNAI3-AS1, AC023024.1, LINC01561, and CYTOR were identified as protective factors, while H19, AC092718.4, LINC01503, and LINC00092 were identified as risk factors (**Figure 1I**). To further elucidate the functional relationships and cross-talk between pathways amongthe 21 PCD-shared genes identified, we performed protein-protein interaction (PPI) network analysis and pathway cross-talk analysis. The results showed that these genes were significantly enriched in shared upstream transcription factor regulatory networks, including TP53, RELA, and STAT3. At the same time, downstream effector molecules such as CASP3, PARP1, GPX4, and MLKL exhibited significant functional overlap across different PCD pathways. For example, GPX4 is not only a core executor of ferroptosis but also participates in regulating lipid peroxidation during necrotic apoptosis; while CASP3 plays a key role in both apoptosis and pyroptosis. These results indicate that different types of PCD pathways are not independent of one another but rather form an integrated regulatory network for programmed cell death through shared regulatory factors and effector molecules (**Supplementary Figure 1**). This finding provides a biological basis for subsequent integrated multi-pathway lncRNA analysis.

**Table 1:** Prognostic programmed cell death-associated lncRNAs.

id	coef	HR	HR.95L	HR.95H	Pvalue
H19	0.272486	1.313225	1.11756	1.543147	0.000932
LINC01503	0.637701	1.892126	1.171296	3.056561	0.009157
AC092718.4	0.570228	1.768671	1.038439	3.012401	0.035835
AC023024.1	-1.18264	0.30647	0.13494	0.696038	0.004717
LINC00092	0.648596	1.912853	1.299721	2.815224	0.001004
SNAI3-AS1	-2.20378	0.110386	0.047232	0.257981	3.62E-07
LINC01561	-0.63684	0.528964	0.341092	0.820314	0.004445
CYTOR	-0.45379	0.635218	0.421764	0.9567	0.02987

### Building the risk model

**Figure 2:**
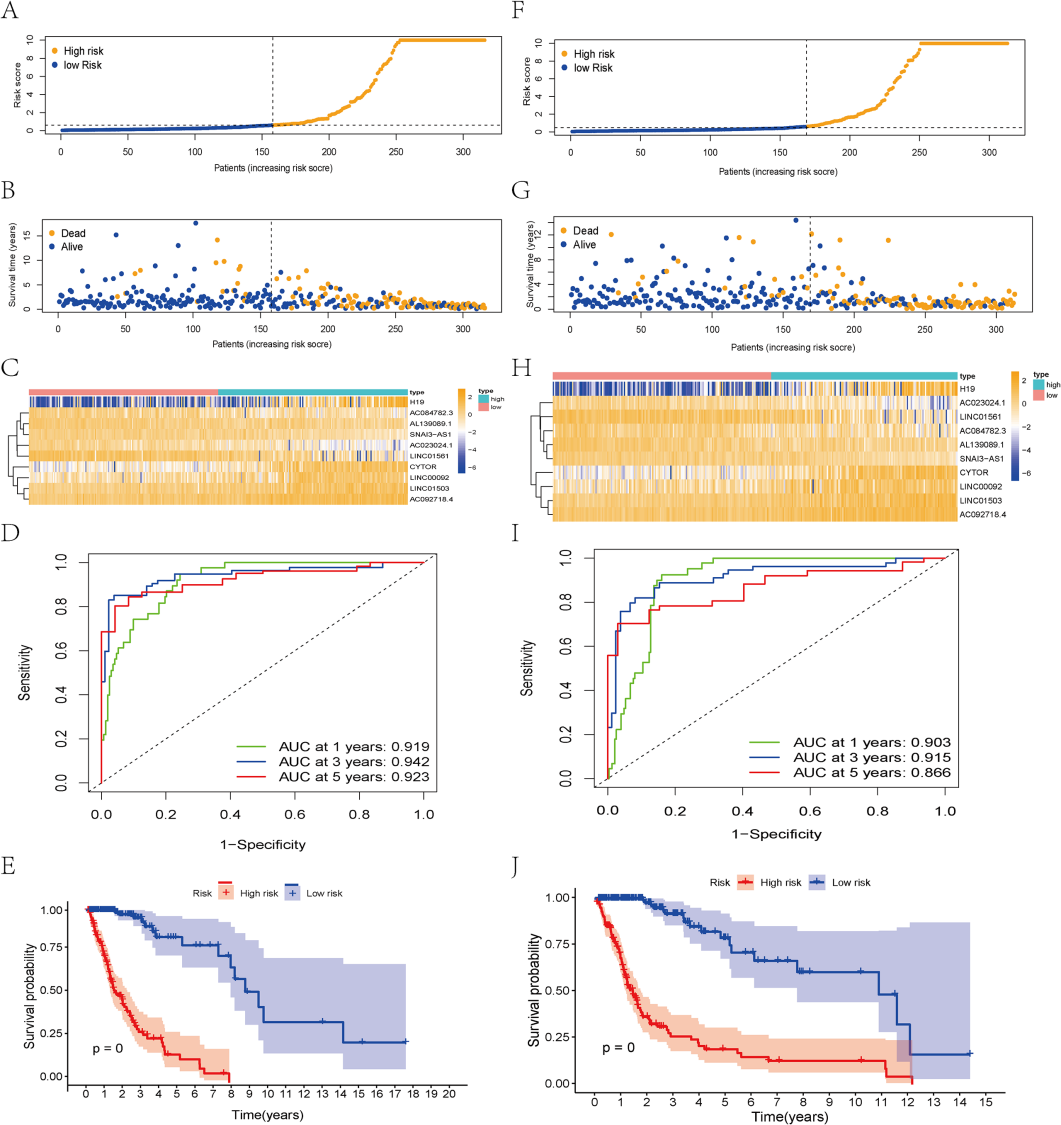
Construction and validation of the programmed cell death-associated lncRNAs signature for survival prediction. **(A-E)** Distribution of RS; survival time and status of patients; heatmap of programmed cell death-associated lncRNAs of RS; ROC curve; K-M curve for training Data. **(F-J)** Distribution of RS; survival time and status of patients; heatmap of programmed cell death-associated lncRNAs of RS; ROC curve; K-M curve for validation Data.

We use a scoring formula to calculate each patient’s risk score, specifically: RS = (0.272 * H19 expression) + (0.637 * LINC01503 expression) + (0.570 * AC092718.4 expression) + (-1.182 * AC023024.1 expression) + (0.648 * LINC00092 expression) + (-2.203 * SNAI3-AS1 expression) + (-0.636 * LINC01561 expression) + (-0.453 * CYTOR expression). Patients were categorized into two groups based on their RS scores (**Figures 2A, B**). Heatmaps revealed significant differences in lncRNA expression levels (**Figure 2C**). The AUC values for the training set data were 0.91 (1 year), 0.94 (3 years), and 0.92 (5 years). The K-M curves similarly indicated that patients with high-risk scores had poorer outcomes (**Figures 2D, E**). Subsequently, the study reveals that the validation set results align with those of the training set: high-score patients showed a positive correlation with poorer prognosis (**Figures 2F, G**). LncRNA expression levels in the validation set were similar to those in the training set and exhibited high AUC values (**Figures 2H, I**). K-M analysis further validated the model’s predictive performance (**Figure 2J**).

### Construction of nomogram

**Figure 3:**
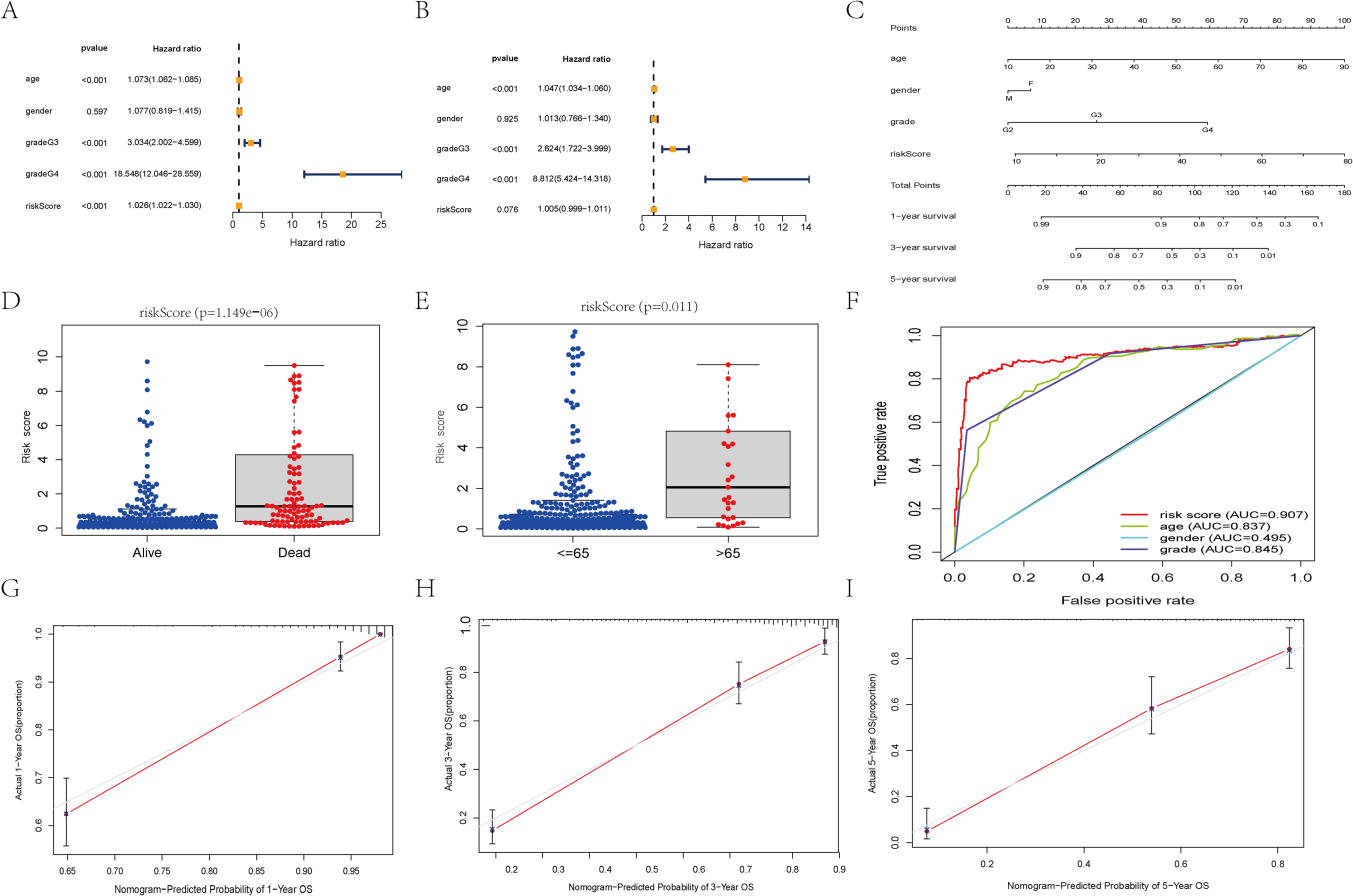
Independent prognosis analysis of risk score. **(A)** Univariate COX Forest plot of risk score. **(B)** Multivariate COX Forest plot of risk score. **(C)** A nomogram based on prognostic features. **(D, E)** Relationship between age, survival status, and risk score of patients. **(F)** ROC curve of risk score and clinical characteristics. **(G-I)** Calibration plots of the nomogram for predicting the probability of OS at 1, 3, and 5 years.

To facilitate clinical practice, we created a nomogram based on age, sex, grade, and risk score. Furthermore, the risk score serves as an independent predictor of overall survival in glioma patients (**Figures 3A, B**). Subsequently, we constructed the nomogram based on clinical-pathological data and risk scores (**Figure 3C**). Further analysis revealed significant correlations between the risk model and patient age and prognosis (**Figures 3D, E**). The ROC curve indicated that the predictive risk model demonstrated superior performance compared to other clinical data, with an AUC value of 0.907 (**Figure 3F**). The model’s reliability was also validated through calibration curves (**Figures 3G–I**).

**Figure 4:**
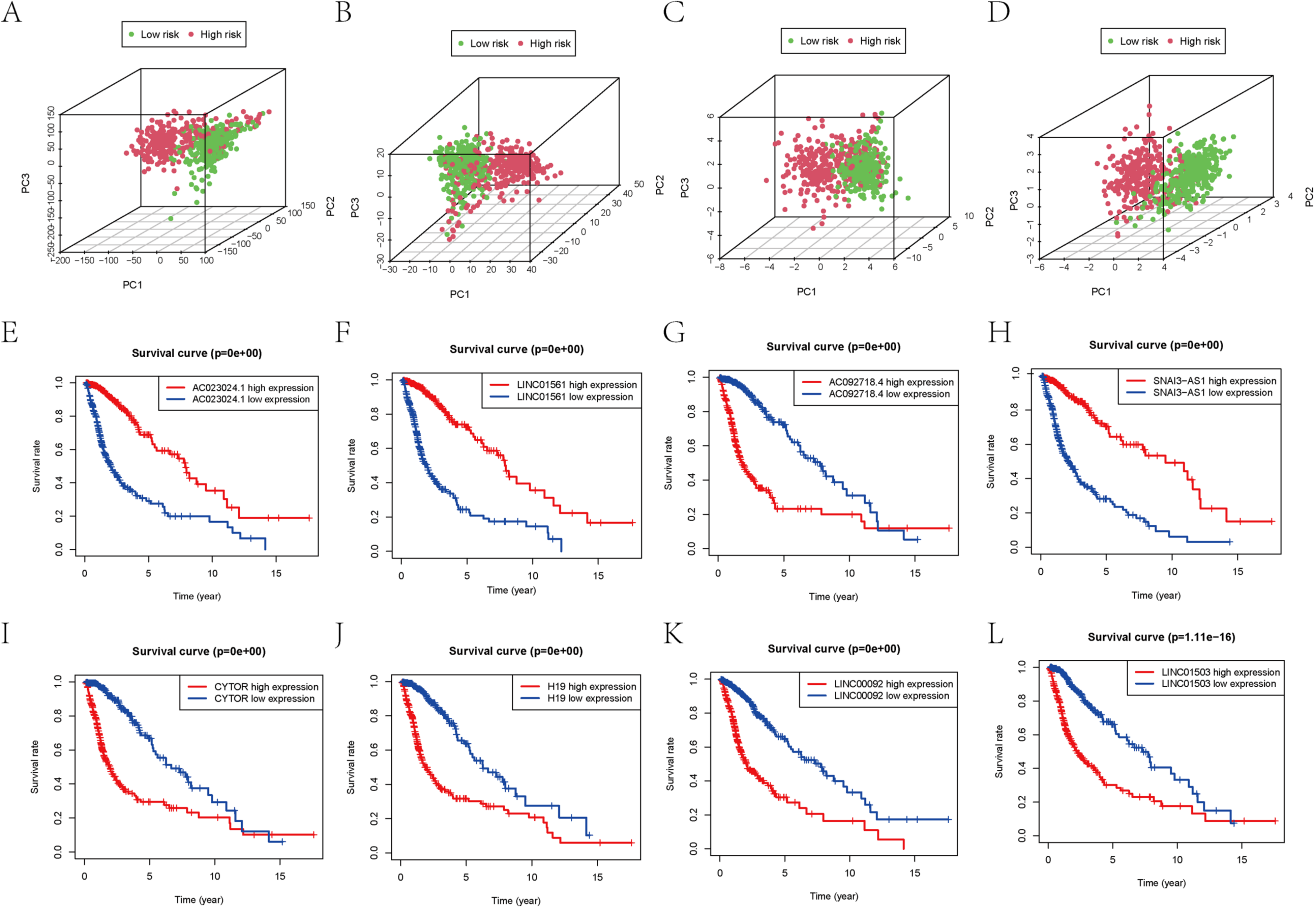
PCA maps of the glioma dataset show the distribution of patients based on the **(A)** whole genome; **(B)** programmed cell death-associated gene sets; **(C)** programmed cell death-associated lncRNAs; and **(D)** prognostic programmed cell death-associated lncRNAs. **(E-L)** K-M analysis of lncRNA expression level in predictive model and prognosis of patients.

To further validate the model’s reliability, PCA analysis demonstrated that two group patients could be clearly separated based on the risk model’s predictors (**Figures 4A–D**). Subgroup analysis subsequently revealed that risk scores predicted outcomes across different patient cohorts (**Figures 4E–L**). K-M curve results indicated that high expression of H19, AC092718.4, LINC01503, and LINC00092 correlated with poor prognosis, whereas patients with elevated levels of SNAI3-AS1, AC023024.1, LINC01561, and CYTOR demonstrated better outcomes. These findings collectively reinforce the predictive potential of the RS.

### Immune function analysis of predictive models

**Figure 5:**
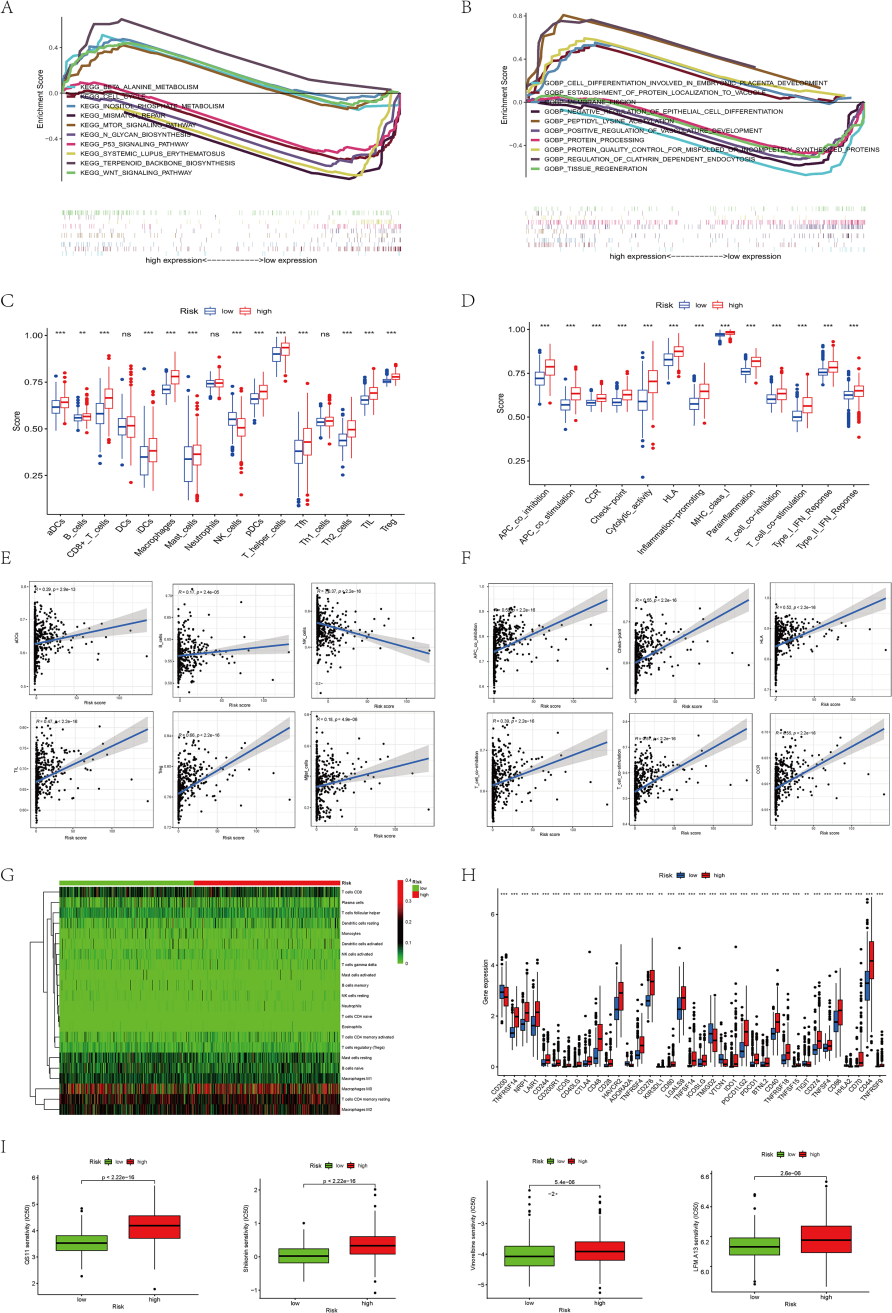
Functional enrichment analysis of 8 prognostic programmed cell death-associated lncRNAs. **(A)** KEGG analysis of 8 prognostic programmed cell death-associated lncRNAs. **(B)** GO analysis of 8 prognostic programmed cell death-associated lncRNAs. **(C)** Analysis of immune cell infiltration levels showed that aDCs, B cells, CD8+ T cells, DCs, macrophages, mast cells, neutrophils, helper T cells, Th1 cells, TILs, and Tregs were expressed at higher levels in the high-risk group. **(D)** Analysis of immune pathways revealed that APC-related pathways, CCR, checkpoint, HLA, MHC class, and T-cell-related pathways were more significant in the high-risk group. **(E)** Correlation analysis between immune cells and risk scores showed that aDCs, B cells, TILs, Tregs, and mast cells were positively correlated with risk scores, while NK cells were negatively correlated with risk scores. **(F)** Correlation analysis between immune pathways and risk scores showed that APC co-inhibition, checkpoint, HLA, T-cell co-inhibition, T-cell co-stimulation, and CCR were positively correlated with risk scores. **(G)** Analysis of immune cell infiltration between the high-risk group and the low-risk group. **(H)** Analysis of immune checkpoints between the high-risk group and the low-risk group. **(I)** IC50 of QS11, Shikonin, Vinorelbine, and LFM.A13 in high and low-risk groups. (ns *P* > 0.05, *P< 0.05; **P< 0.01; ***P< 0.001).

Recently, immunotherapy for gliomas has become a research spotlight. Therefore, we explored the immune-related functions of lncRNAs via GSEA and found them primarily enriched in immune-related pathways and tumorigenesis-associated pathways (**Figures 5A, B**). Further ssGSEA analysis revealed that most cell types (CD8^+^ T, macrophages, helper T, TILs) were significantly heightened in the high-risk group (**Figures 5C, D**). The specific ratios and correlation coefficients between immune pathways and cells were also presented (**Figures 5E, F**). We further analyzed common immune checkpoints and found that the high-risk group exhibited higher expression levels of IDO1, CD40, and CD70 in the high-risk cohort, while CD200 was higher in the low-risk cohort (**Figures 5G, H**). Finally, the associated drug prediction analysis revealed higher IC50 values for QS11, Shikonin, Vinorelbine, and LFM.A13 in the high-risk cohort (**Figure 5I**). However, although the above analyses of the relationship between lncRNAs and immune cells and pathways have yielded promising results, they remain speculative at this stage and will require further validation through detailed basic experiments in the future.

### AC092718.4 effects on gliomas and immunological analysis

**Figure 6:**
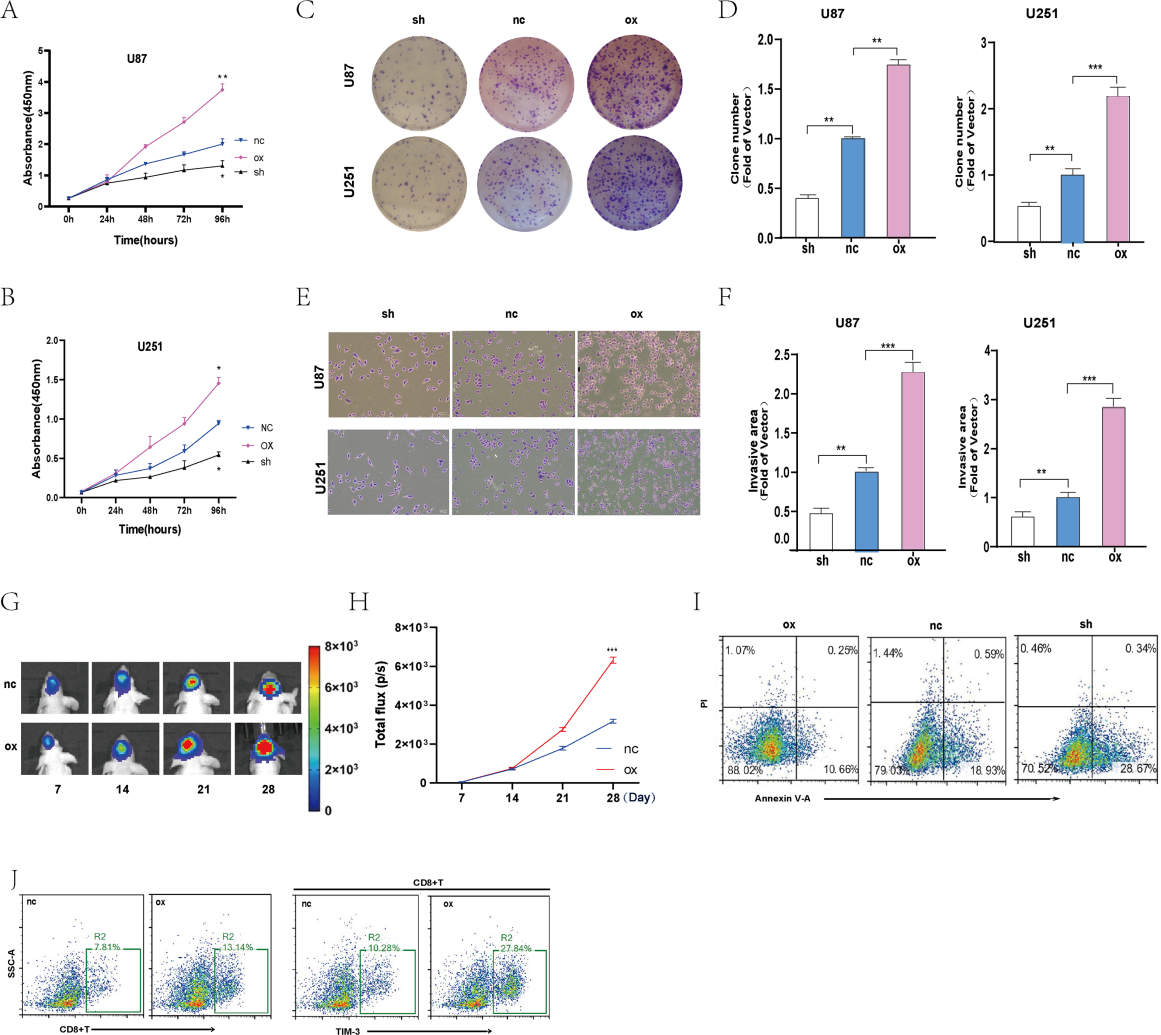
Overexpression of the AC092718.4 gene can enhance the proliferation and invasion of U87 and U251 cells. **(A, B)** CCK8 cell experiment to detect the proliferation of U87 and U251 cells. **(C, D)** Cell cloning to detect the survival of U87 and U251 cells. **(E, F)** The infiltration capacity of U87 and U251 cells was assessed using the Transwell assay. **(G, H)** *In vivo* imaging of intracranial tumor growth was performed in nude mice. **(I)** Flow cytometry was used to assess the effect of AC092718.4 on apoptosis. **(J)** Flow cytometry analysis of immune cells in intracranial tumors in the animal model. (*P<0.05; **P<0.01; ***P<0.001).

Among the predicted factors in the model, other lncRNAs besides AC092718.4 have been studied in central nervous system tumors. Therefore, we decided to investigate its biological function in subsequent cellular and animal experiments. First, we established two glioblastoma cell lines (U87 and U251) that overexpress AC092718.4. Next, in cellular functional assays, we employed the CCK8 assay to evaluate the impact of AC092718.4 on cell proliferation. Results showed no significant changes across groups within 48 hours. After 96 hours, the proliferation capacity of glioma cells overexpressing AC092718.4 was markedly enhanced (**Figures 6A, B**). The cell clonogenic assay demonstrated that overexpression of AC092718.4 increased glioma cell survival rates, with statistically significant differences (**Figures 6C, D**). Next, we examined the cell invasion capacity and found that overexpression of AC092718.4 significantly enhanced the invasive ability (**Figures 6E, F**). Next, we performed CCK-8 assays, as well as assays for cell cloning and invasion capacity, on U87 and U251 cells with AC092718.4 knockdown using the same method. The results revealed the opposite conclusion: AC092718.4 knockdown inhibited the proliferation, colony formation, and invasion capacity of glioma cells.

To further test the functional effects of AC092718.4 on glioma, we established an *in situ* intracranial glioma animal model to investigate its biological effects. Following tumor establishment in mice, weekly *in vivo* imaging was performed to monitor tumor growth and plot intracranial tumor growth curves. Fluorescence values for intracranial tumors in both nude mouse groups showed no significant differences during the first 14 days post-cell injection. Tumor fluorescence values began to diverge at day 21. After 28 days, tumors overexpressing AC092718.4 exhibited significantly higher fluorescence values than the control group (**Figures 6G, H**). These findings suggest that AC092718.4 overexpression enhances the biological properties of gliomas, including proliferation and invasion. To investigate the relationship between AC092718.4 and specific programmed cell death pathways, we conducted apoptosis assays in three U87 cell lines, including AC092718.4 knockdown, overexpression, and control groups. The results showed that, compared with the control group, the proportion of apoptotic cells was significantly reduced in the AC092718.4 overexpression group, while it was significantly increased in the knockdown group. These findings indicate that AC092718.4 promotes glioma progression by inhibiting apoptosis (**Figure 6I**).

Finally, flow cytometry analysis of tumor-infiltrating immune cells revealed that, compared with the control group, the proportion of CD8^+^ T cells infiltrating tumor tissue was significantly higher in the AC092718.4 overexpression group. Concurrently, the expression level of TIM-3 on the surface of CD8^+^ T cells in this group was increased, suggesting that tumor tissues in the AC092718.4 overexpression group exhibited a more pronounced T-cell exhaustion phenotype. This indicates that high expression of AC092718.4 may be associated with enhanced immune cell infiltration and the formation of an immunosuppressive microenvironment (**Figure 6J**). Furthermore, these findings explain why CD8^+^ T cell expression levels were significantly higher in the high-risk group compared to the low-risk group in the immune cell analysis.

## Discussion

Glioma, which accounts for about 81% of malignant central nervous system tumors, is the most common primary brain malignancy (26). Even with significant progress in its management over the past years, patient outcomes remain poor (27, 28). Consequently, exploring new molecular targets and personalized therapeutic approaches is urgently required.

Extensive research has revealed that PCD is a genetically encoded biological process playing a central role in heredity, development, and disease (29). During normal heredity and development, PCD shapes organ morphology, optimizes neural network connections, and maintains tissue homeostasis by precisely eliminating specific cells, thereby ensuring normal individual development and internal environmental stability (30). In recent years, research on selectively inducing tumor cell death through PCD mechanisms to inhibit tumor growth and metastasis has emerged as a significant research focus (31). Given the widespread occurrence of apoptosis in tumors, targeting its associated pathways has become a therapeutic strategy for cancer treatment. Currently, a successful example of targeting apoptosis pathways for cancer therapy—Venetoclax—has been used to treat CLL and AML through highly selective and potent inhibition of the BCL-2 protein (32). Similarly, these drugs have shown potential in studies of other solid tumors, such as SCLC, MM, and lymphoma (33, 34). Meanwhile, owing to the autophagy function of tumor cells, this makes it a candidate therapeutic target for intervening in pancreatic cancer (35), BC (36), and NSCLC (37). On the other hand, ferroptosis, as a cell death pathway gaining significant attention, is increasingly recognized for its potential in antitumor therapy, particularly against tumor cells resistant to conventional apoptotic pathways. In many cancer types, for example HCC, pancreatic cancer, and GBM, tumor cells exhibit heightened sensitivity to ferroptosis, making it a promising new therapeutic target (38). Moreover, recent studies have demonstrated that lncRNAs play diverse roles in tumor growth and metastasis (39–41). However, most current functional studies of PCD-associated lncRNAs in glioblastoma patients focus on a single type of PCD, failing to comprehensively examine this vital physiological process as a unified entity. Consequently, previous findings may exhibit certain biases, which limit the ability to explore PCD’s critical role in glioma fully. Therefore, this study aims to identify lncRNAs commonly associated with PCD, construct predictive risk models, and elucidate the evolutionary and immunological functions of these lncRNAs.

This study integrated lncRNAs associated with various PCD pathways—including apoptosis, pyroptosis, ferroptosis, necroptosis, and autophagy—to construct a prognostic model. It should be noted that this integrative strategy does not simply involve the mechanical superimposition of different biological processes, but is instead grounded in a clear biological foundation. Recent studies have confirmed that extensive molecular interactions and functional compensation exist among different PCD pathways (15). For example, when the apoptosis pathway is inhibited, tumor cells may switch to ferroptosis or necrotic apoptosis to evade death. For example, when the apoptotic pathway is inhibited, tumor cells may switch to ferroptosis or necrotic apoptosis to evade cell death (42); conversely, the loss of GPX4, a key molecule in ferroptosis, can also activate the apoptotic pathway (43). This phenomenon of “crosstalk” and “compensation” between pathways is particularly pronounced in gliomas, which partly explains why therapeutic strategies targeting a single PCD pathway often yield limited results. Therefore, screening for shared lncRNAs from the perspective of multi-pathway synergistic regulation better reflects the state of cell death regulation in tumor cells within their actual microenvironment. The results of this study also support this view: the eight characteristic lncRNAs identified were not enriched in a single type of PCD pathway but were simultaneously mapped to multiple death pathways, further validating the rationale and necessity of integrated analysis.

First, this study identified 21 genes commonly expressed across different types of programmed cell death. Subsequently, bioinformatics analyses were used to determine 36 predictive features of lncRNAs associated with programmed cell death. Analysis identified SNAI3-AS1, AC023024.1, LINC01561, and CYTOR as protective factors; the rest are the opposite. Then, we utilized these eight predictors to construct a risk model. Previous studies have consistently reached the following conclusions regarding these predictors: SNAI3-AS1 acts as a protective factor, enhancing erastin’s antitumor activity by promoting ferroptosis, and inducing ferroptosis to improve glioma outcomes (44). Silencing LINC01561 affects survival, migration, and proliferation in glioma cells (45). In oral squamous cell carcinoma (OSCC), LncRNA CYTOR drives aberrant glycolysis by stabilizing ZEB1 through an HNRNPC-mediated mechanism, which consequently suppresses tumor proliferation and metastasis (46). The lncRNA H19 has attracted considerable research attention in recent years owing to its widespread ectopic expression across diverse tumors. Accumulating evidence indicates that H19 expression is linked to risk factors in a spectrum of cancers, including OSCC, HCC, BC and colorectal cancer (47). Similarly, AC092718.4 has been recognized as a risk factor in the context of both lung adenocarcinoma (LUAD) and BC (48). Furthermore, diminished expression of LINC00092 is correlated with tumorigenesis and metastatic progression in LUAD, and its loss is associated with unfavorable patient prognosis (49). LINC01503 enhances tumor malignancy by promoting cancer stemness in glioblastoma cells (50). Thus, the predictive factors identified in this study demonstrate high reliability and practical utility.

Next, patients were stratified into two groups to functionally explore predictive factors, and their roles in immune function were evaluated through enrichment analysis and immune-infiltration analysis. We identified a correlation between the model’s predictive factor “cell cycle” and tumor immunity. GSEA analysis revealed its enrichment in pathways including MISMATCH-REPAIR, MTOR-SIGNALING-PATHWAY, P53-SIGNALING-PATHWAY, and SYSTEMIC-LUPUS-ERYTHEMATOSUS. Further investigation of immune cells and pathways revealed that, beyond NK cells, aDCS, CD8^+^ T, Th1 cells, TLI, and Tregs were more prevalent in individuals at high risk. Immune pathways, including Checkpoint, HLA, and T-cell, were significantly more pronounced in this group. Previous studies have similarly revealed that glioma cells secrete a diverse array of chemokines and cytokines. These substances enhance infiltration of astrocytes, endothelial cells, and immune cells (macrophages, MDSCs), CD4^+^ T, and Treg cells. Identifying these factors may aid in improving glioma immunotherapy by targeting immune regulation and immune escape mechanisms (51–53). Peggs et al. also observed elevated CTLA-4 expression in high-grade gliomas, which correlates with poorer prognosis (54). Furthermore, studies have shown that CD70 is overexpressed in both primary and recurrent gliomas and correlates with lower survival rates. This may result from CD70 inducing T cell exhaustion or apoptosis in tumor cells, particularly CD8^+^ T cells, while activating regulatory T cells to mediate immunosuppression (55, 56). Our research on immune cell infiltration also confirms this: although the proportion of CD8^+^ T cells infiltrating tumor tissue is significantly elevated, the majority exhibit an exhausted phenotype and are, on the whole, in an immunosuppressed state. Research on CD276 has also revealed its widespread overexpression on tumor cells and the tumor vasculature. It serves as a GBM risk marker by mediating immune suppression through the inhibition of NK cell activity and the induction of tumor cell invasion and differentiation (57, 58).

Finally, through glioma cell and animal experiments, the impact of lncRNA AC092718.4 on glioma cell viability, proliferation, and invasive capacity was further validated within the risk prediction model, thereby enhancing the model’s reliability. Results indicate that the screened PCD-associated lncRNA holds potential for predicting glioma patient prognosis and offers novel insights for personalized therapy. However, the study retains limitations, as the precise mechanism by which AC092718.4 regulates PCD remains unclear, necessitating further experimental exploration of its role in promoting tumor progression via PCD. The lack of independent external cohorts, such as the CGGA, is one of the limitations of this study. Internal validation (even with complex resampling) may still overestimate the model’s performance in real-world populations. Therefore, before this model can be widely applied, it is imperative to conduct external validation in larger, multicenter, prospective cohorts. Furthermore, this study has not yet thoroughly investigated the pathways or mechanisms through which AC092718.4 exerts its tumor-promoting effects in specific PCD pathways; future research should combine loss-of-function models with multidimensional PCD assays to further elucidate its molecular mechanisms. Future research will expand sample sizes and strengthen experimental validation to elucidate the specific functions and mechanisms of this phenomenon.

In summary, this study delineated a set of lncRNAs with significant predictive value in programmed cell death. These findings not only carve out novel perspectives for developing cancer therapeutic strategies but also illuminate new avenues for foundational research on lncRNAs in this critical cellular process.

## Data Availability

The datasets presented in this study can be found in online repositories. The names of the repository/repositories and accession number(s) can be found in the article/**Supplementary Material**.
