# Supplementary figures and images for: Comprehensive exploration of the role of multimodal programmed cell death- associated lncRNAs in the prognosis and immunity of glioma
Source: Front Immunol. 2026 May 14;17:1765882. doi: 10.3389/fimmu.2026.1765882 (PMC13215893; doi:10.3389/fimmu.2026.1765882)

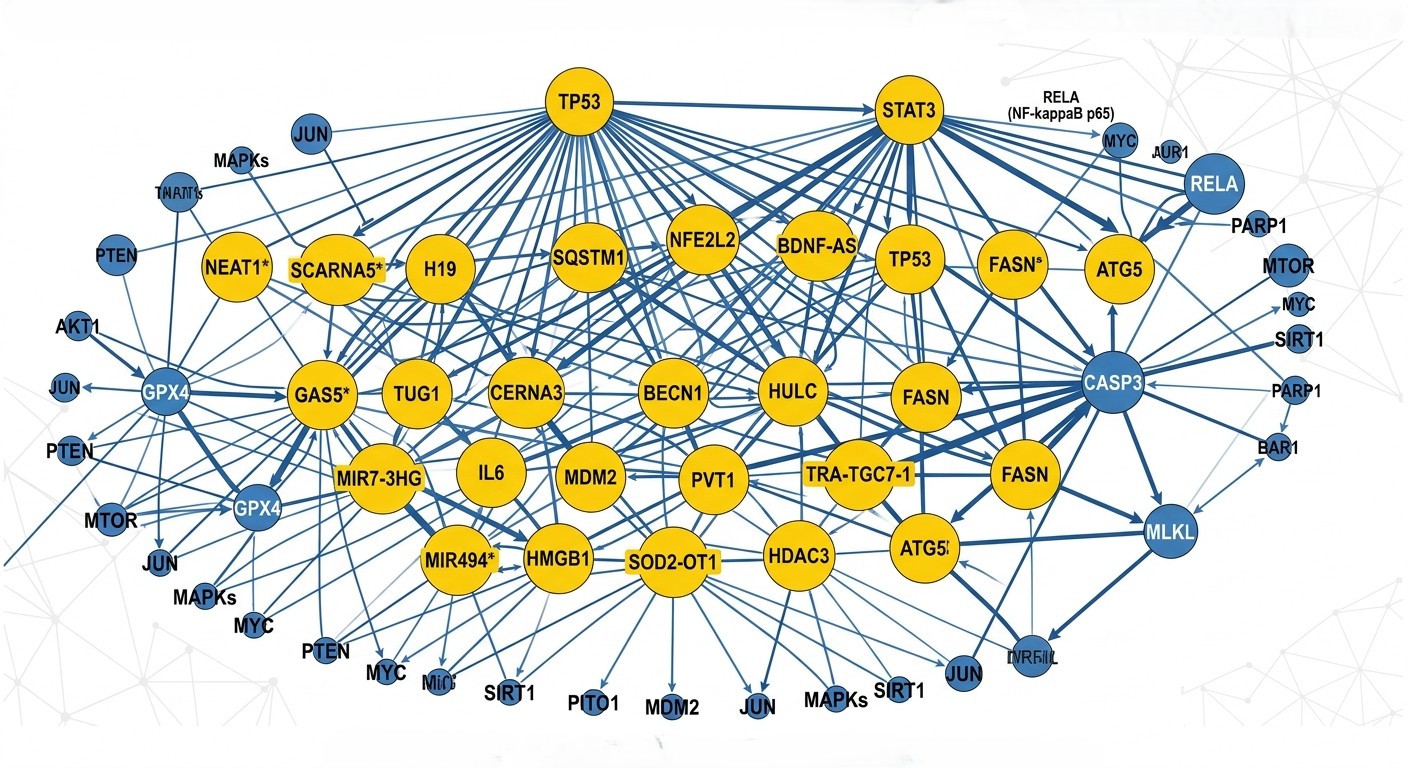

Supplement: Supplementary file 1 [file Image1.jpeg]
